# Emergency radiology of severe acute injury in extreme alpine summer sports: a pictorial essay

**DOI:** 10.1007/s10140-026-02508-9

**Published:** 2026-07-01

**Authors:** Milena Calabrese, Federica Vaccarino, Rossana Franciosi, Carlo Cosimo Quattrocchi, Marco Parillo

**Affiliations:** 1 Department of Radiological Sciences and Medical Imaging, Azienda Sanitaria Universitaria Integrata del Trentino (ASUIT) Provincia Autonoma Di Trento, Trento, Italy; 2 Centre for Medical Sciences - CISMed, University of Trento, Trento, Italy

**Keywords:** Athletic Injuries, Emergency Medicine, Diagnostic Imaging, Tomography, X-Ray Computed, Magnetic Resonance Imaging, Multiple Trauma.

## Abstract

Extreme alpine summer sports are increasingly popular and expose participants to high-energy trauma in remote mountain environments. This pictorial essay reviews the emergency radiology of severe acute injuries associated with down-hill mountain biking, rock climbing, paragliding, BASE jumping, canyoning, and rafting, with emphasis on mechanism-based imaging interpretation. Injury patterns differ across disciplines but are largely determined by velocity, fall height, landing posture, impact surface, axial loading, torsional forces, and water-related hazards. Down-hill mountain biking commonly produces extremity, thoracic, craniofacial, abdominal, and spinal injuries after high-speed crashes or handlebar impact. Rock climbing and paragliding frequently involve falls from height and hard landings, leading to thoracolumbar fractures, pelvic trauma, calcaneal or ankle injuries, and, in severe cases, neurologic compromise or visceral injury. BASE jumping is associated with high-velocity landing trauma, particularly involving the lower limbs, pelvis, and spine. Canyoning and rafting combine blunt trauma with environmental risks such as submersion, aspiration, hypothermia, and drowning-related complications. In high-energy trauma or polytrauma, whole-body computed tomography is the cornerstone of emergency assessment, enabling rapid evaluation of head, spine, chest, abdomen, and pelvis. Radiography remains useful for isolated appendicular injuries, whereas magnetic resonance imaging is essential for spinal cord, ligamentous, marrow, and selected soft-tissue injuries. Awareness of sport-specific mechanisms helps radiologists optimize image selection, maintain an appropriate search pattern, recognize injuries that may coexist after axial loading, and communicate urgent findings promptly. A mechanism-oriented approach may improve diagnostic accuracy, guide multidisciplinary management, and reduce missed injuries in patients presenting after alpine summer extreme sports trauma.

**Graphical Abstract:**

**Figure:**
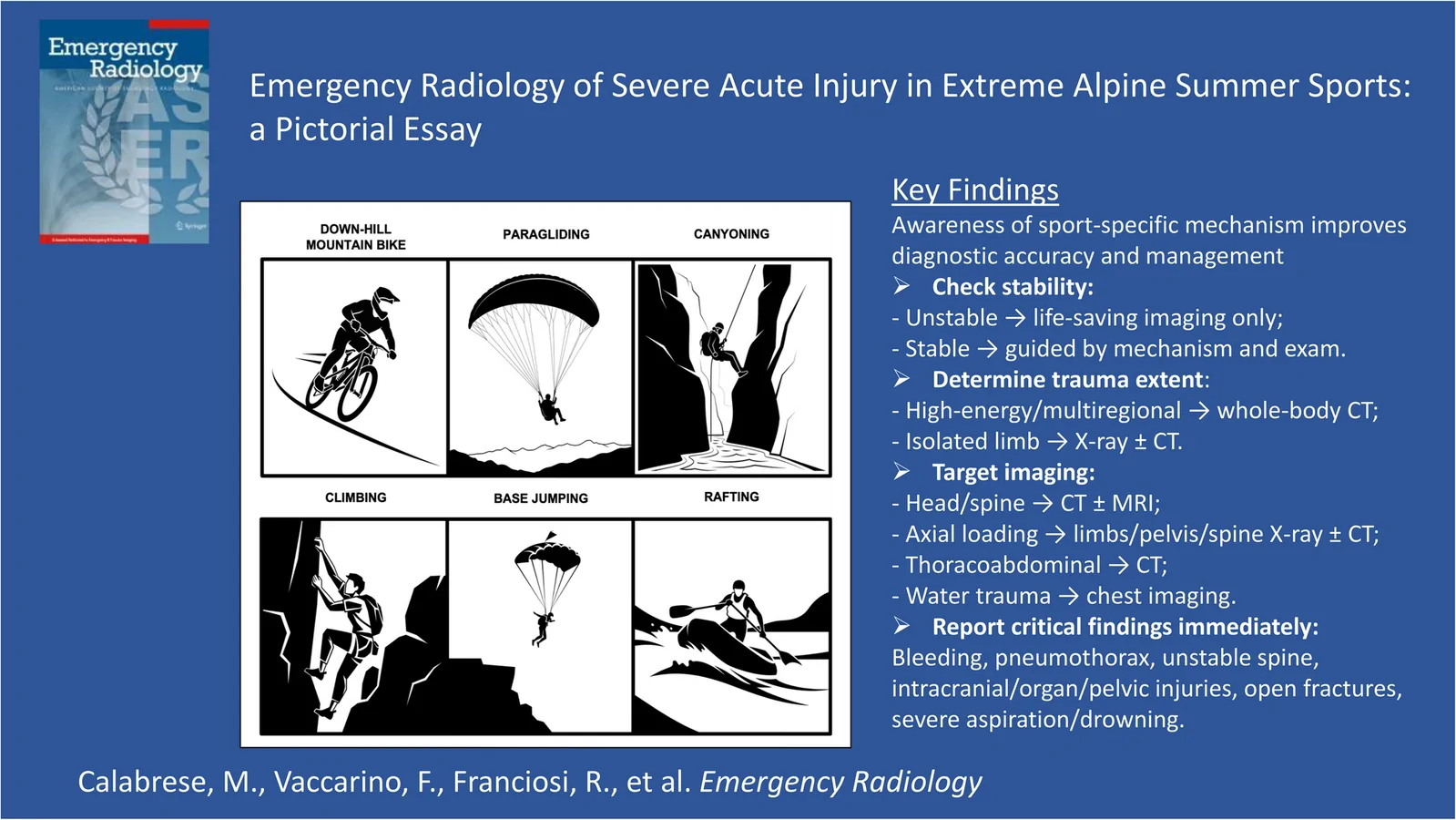

## Introduction

**Fig. 1:**
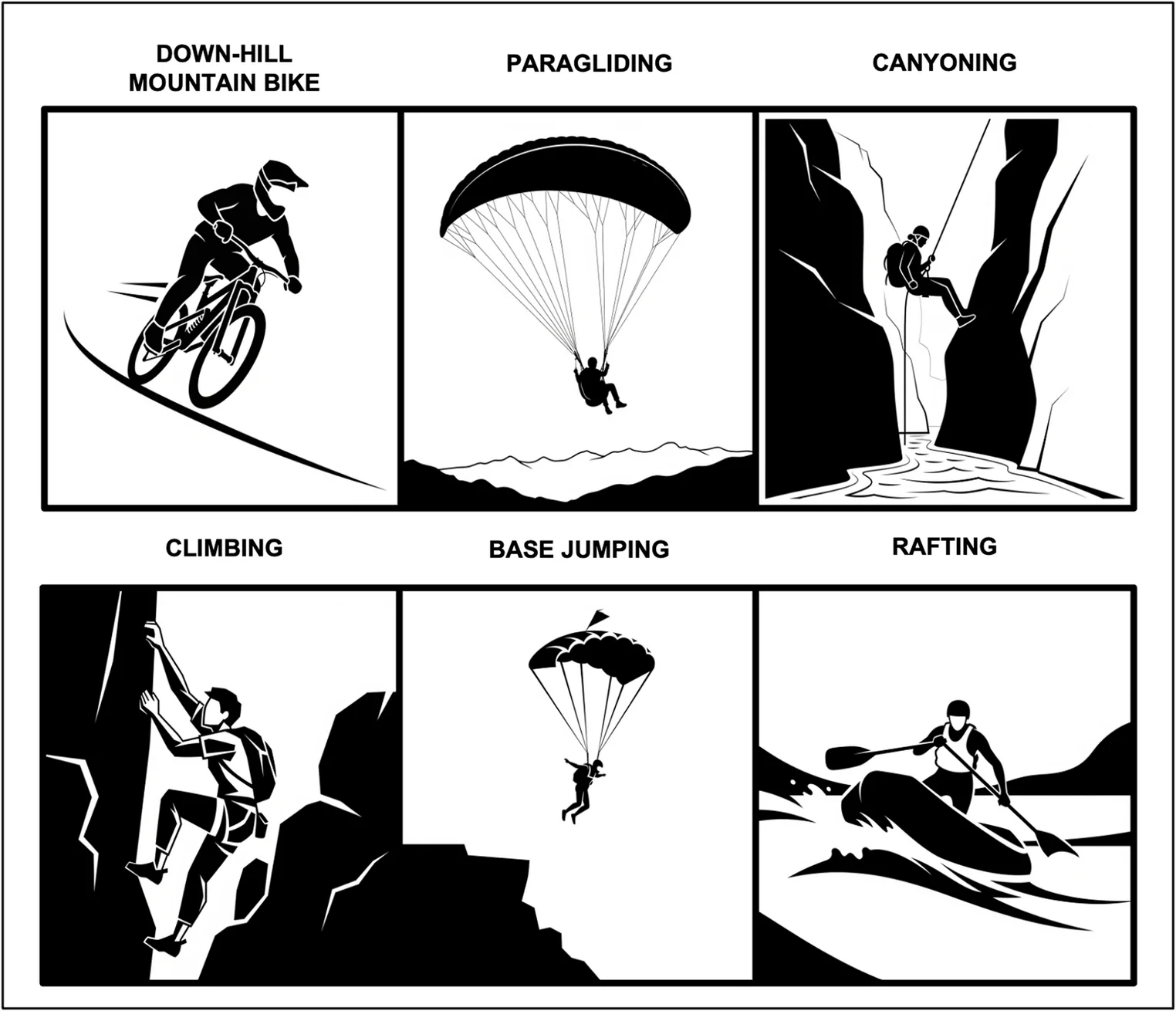
Overview of the six extreme alpine summer sports included in this pictorial essay: down-hill mountain biking, paragliding, canyoning, climbing, BASE jumping and rafting

Alpine regions are recognized for winter sports, but in summer they attract tourists practicing extreme sports (ESs) [1]. Down-hill mountain biking (DH-MTB), rock climbing, paragliding, BASE (Buildings, Antennas, Spans, Earth) jumping, canyoning, and rafting (Fig. 1) have increased in popularity, driven by accessibility and the search for high-adrenaline experiences [2, 3]. Although canyoning and rafting are not exclusively alpine activities, they are included in this pictorial essay because, in alpine regions, they are commonly performed in mountain rivers, gorges, waterfalls, and narrow valleys, where steep terrain, cold water, rock impact, remoteness, and delayed rescue may influence both injury patterns and emergency imaging priorities [4–8]. ESs expose participants to acute trauma mechanisms that differ from conventional recreational injuries because they frequently combine velocity, vertical height, abrupt deceleration, axial loading, torsional forces, direct impact against irregular surfaces, and water-related hazards [3, 4]. Injuries may be multisystemic, clinically occult at presentation, or underestimated if imaging is not tailored to the mechanism of trauma [9].

Radiology plays a crucial role in the rapid diagnosis and management of injuries associated with ESs [10].The radiologist’s role is not limited to confirming the obvious fracture or hemorrhage, but includes anticipating associated injuries according to mechanism, selecting the appropriate imaging pathway, and promptly communicating findings that may change acute management [9, 10].

The aim of this pictorial essay is to provide radiologists with a mechanism-based and injury-pattern-oriented imaging framework for severe acute trauma related to alpine summer ESs. After reading this article, the reader should be able to: (1) recognize the main trauma mechanisms associated with each sport; (2) anticipate the body regions and associated injuries that require active search; (3) select an appropriate imaging strategy according to clinical severity; and (4) communicate time-critical findings that may influence patient management.

## Imaging approach in extreme sport trauma

In alpine summer ES trauma, imaging should not be guided only by the most evident painful region, but by the combination of clinical severity, mechanism of injury, and expected pattern of associated lesions. A mechanism-based approach is particularly important because patients injured during extreme sports may present with focal symptoms despite multiregional trauma. High velocity, abrupt deceleration, vertical impact, axial loading, and direct collision with irregular surfaces can produce clinically occult injuries, especially involving the spine, pelvis, thorax, abdomen, and head [9, 11].

For the purposes of this pictorial essay, “high-energy mechanism” refers to trauma in which the amount or direction of energy transfer raises concern for clinically significant injury beyond a single anatomic region. In alpine summer sports, this includes high-speed downhill biking crashes, over-the-handlebar impact, collision against rocks or trees, hard landings after aerial sports, falls during climbing or canyoning, ejection from a raft, and trauma associated with loss of consciousness, neurologic deficit, respiratory compromise, hemodynamic instability, or pain in multiple body regions [3–9, 12–20]. Similarly, “fall from height” should be considered a mechanism rather than a purely numerical threshold. Injury severity depends not only on the measured height, but also on landing posture, impact surface, velocity, protective equipment, axial loading, and secondary impacts against rock, ground, water, trees, or artificial structures [9, 11, 21]. 

In stable or stabilized patients with suspected major blunt trauma or multiregional injury, whole-body computed tomography (CT) is the key modality because it rapidly evaluates the head, spine, chest, abdomen, and pelvis in a single integrated examination. This approach is particularly relevant when the clinical examination is unreliable, when the mechanism is high energy, or when injuries in different body regions may coexist after axial loading or deceleration [22–25].

Radiography remains appropriate for stable patients with isolated appendicular trauma and low suspicion of multisystem injury. However, CT should be used when fracture anatomy is complex, intra-articular, radiographically occult, or relevant for operative planning, and when the mechanism suggests associated injuries outside the field of the initial radiographs [10, 11, 22].

Magnetic resonance imaging (MRI) has a complementary role and is usually performed after initial stabilization rather than during the primary assessment of unstable trauma. Its main indications include suspected spinal cord injury, ligamentous disruption, epidural hematoma, marrow edema, traumatic axonal injury, and neurologic symptoms that are unexplained or underestimated by CT [21, 22, 26].

Water-related ES trauma requires additional attention because traumatic and non-traumatic complications may coexist. In canyoning and rafting, blunt impact injuries may be associated with submersion, aspiration, hypothermia, near-drowning-related pulmonary complications, and hypoxic brain injury, so imaging should be guided by both trauma severity and respiratory or neurologic status [5–8, 21, 24].

Table 1 translates these principles into a general imaging algorithm for emergency practice. The following sport-specific sections apply this general algorithm to the characteristic mechanisms encountered in alpine summer ESs.

**Table 1:** General imaging algorithm for severe alpine summer extreme-sports trauma. eFAST, Extended Focused Assessment with Sonography in Trauma; CT, computed tomography; MRI, magnetic resonance imaging

Step 1 — Assess hemodynamic status
• Unstable patient: perform only imaging that can immediately guide life-saving management, such as portable chest radiography, pelvic radiography, and eFAST during the primary survey. Proceed to CT only after stabilization or when local trauma workflow permits safe scanning without delaying hemorrhage control. • Stable or stabilized patient: proceed according to mechanism, number of suspected injured regions, and reliability of the clinical examination.
Step 2 — Identify major trauma or suspected multiregional injury
• High-energy mechanism, suspected multiple injuries, unreliable examination, or pain in multiple regions: perform whole-body CT with intravenous contrast, including head, cervical spine, chest, abdomen, pelvis, and multiplanar thoracolumbar spine reconstructions. • Isolated appendicular trauma with low suspicion of multisystem injury: begin with targeted radiography; add CT when fracture anatomy is complex, intra-articular, radiographically occult, or relevant for surgical planning.
Step 3 — Adapt imaging to the dominant injury pattern
• Axial loading or feet-first landing: actively assess the calcaneus, talus, tibial plafond, knee, pelvis, sacrum, and thoracolumbar spine, because combined lower-limb, pelvic, and spinal injuries may coexist. • Head trauma or neurologic impairment: perform non-contrast head CT and cervical spine CT when indicated; add MRI when CT underestimates neurologic severity or traumatic axonal injury is suspected. • Suspected spinal cord, ligamentous, or epidural injury: perform MRI after stabilization, particularly when neurologic findings are unexplained by CT or when surgical planning requires assessment of neural compression. • Thoracoabdominal impact or abrupt deceleration: perform contrast-enhanced CT of the chest, abdomen, and pelvis; add CT angiography or delayed excretory-phase imaging when vascular or urinary tract injury is suspected. • Water-related trauma or near-drowning: evaluate respiratory status and trauma severity; chest radiography or CT may show aspiration-related consolidation, pulmonary edema, contusion, laceration, pneumothorax, or drowning-related complications.
Step 4 — Communicate time-critical findings
Urgent communication is required for active bleeding, tension pneumothorax, unstable spinal injury, spinal canal compromise, intracranial hemorrhage, major solid-organ injury, complex pelvic trauma, vascular injury, open fracture, and severe aspiration or drowning-related complications.

### Downhill mountain biking: high-speed collisions and impact injuries

Among ground-based alpine summer ESs, DH-MTB is dominated by velocity, abrupt deceleration, and direct impact, making the mechanism closer to high-energy cycling trauma than to low-energy recreational bicycle injuries. DH-MTB involves descent on steep, rough, and technical terrain with rocks, roots, jumps, and drops. Falls, collisions, and abrupt deceleration produce frequent traumatic injuries, particularly involving the extremities [12, 13]. Lateral falls commonly affect the shoulder girdle, clavicle, arm, and ribs, whereas forward falls over or onto the handlebars may cause craniofacial, thoracic, abdominal, and upper-extremity trauma [12, 13]. Sudden deceleration can transmit axial and shear forces to the head, spine, pelvis, and limbs [4, 14].

DH-MTB injuries range from minor skin abrasions and contusions to moderate skeletal trauma, most commonly fractures [12, 13]. Severe injuries are less frequent but may include hemorrhagic concussion, vertebral fractures with or without spinal cord injury, and internal organ damage with bleeding [4, 14]. The lower leg and forearm are among the body regions most often affected [12, 13].

**Fig. 2:**
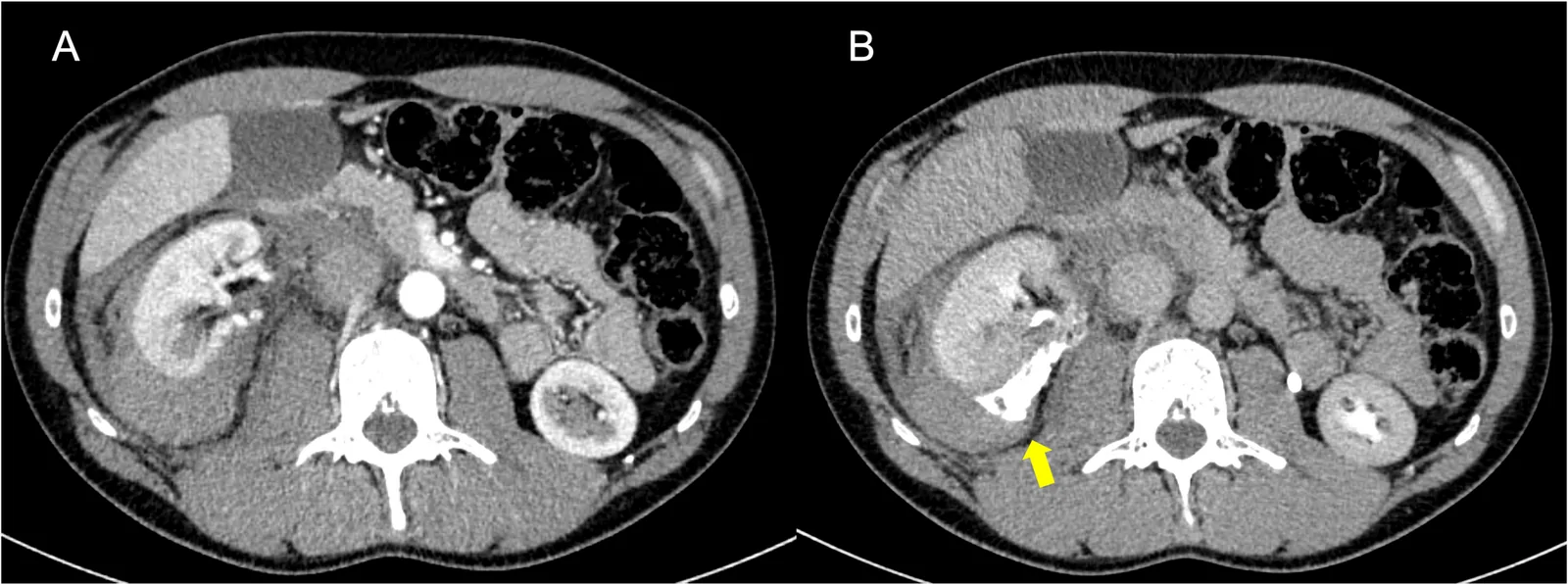
Contrast-enhanced CT images of a 37-year-old patient after a right-sided fall during down-hill mountain biking demonstrate a laceration-contusion injury of the right kidney (**A**), with disruption of the urinary collecting system and extravasation of contrast-opacified urine (yellow arrow, **B**)

**Fig. 3:**
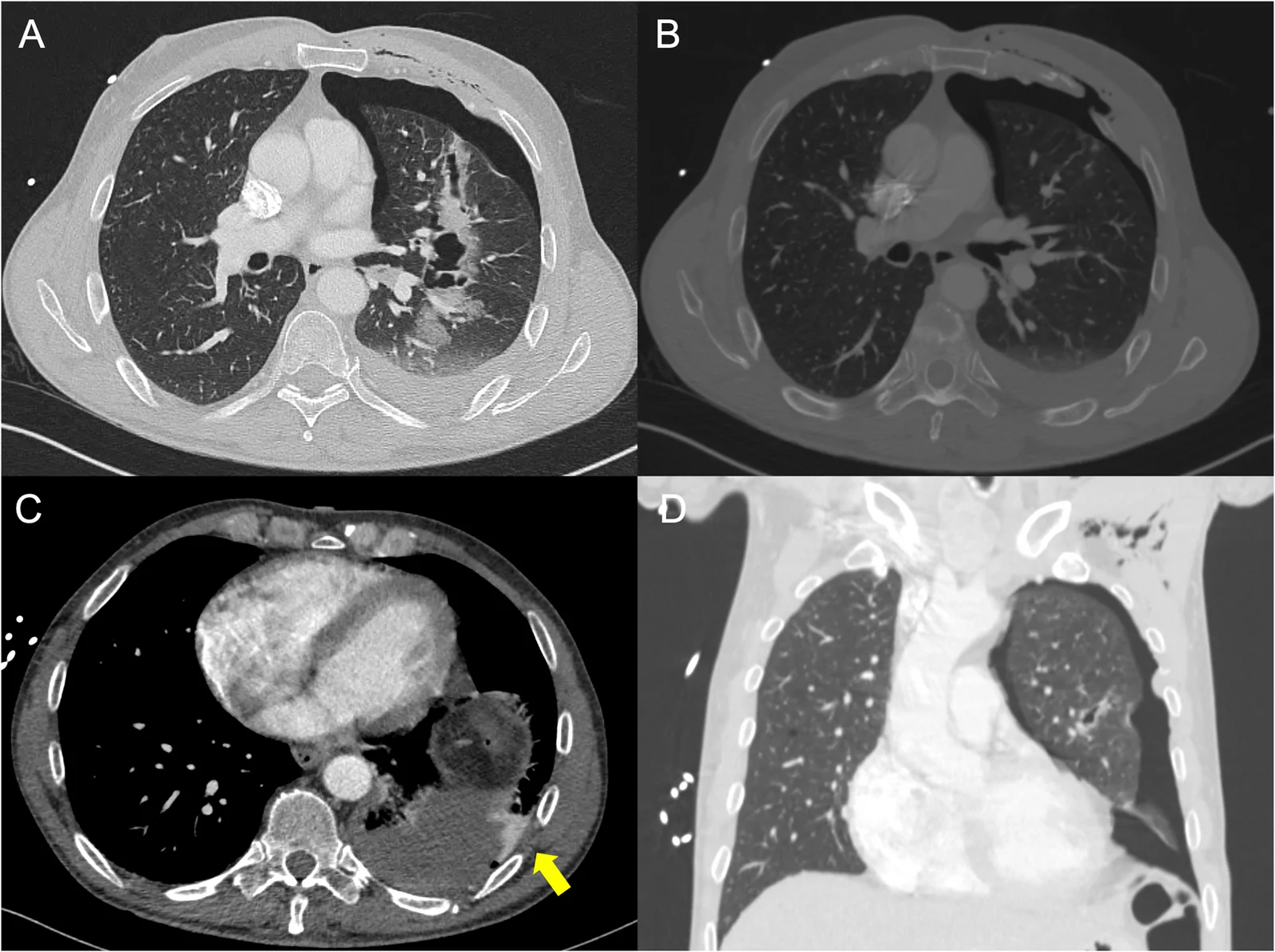
Contrast-enhanced chest CT of a 55-year-old man involved in a high-energy down-hill mountain biking accident, with direct handlebar impact causing a penetrating injury to the left anterior hemithorax. Axial and coronal images show left-sided pneumothorax (**A**, **B**, **D**), rib fractures (**B**), pulmonary laceration-contusion (**A**), and pleural effusion with heterogeneous attenuation, consistent with a partial hematic component (yellow arrow, **C**)

**Fig. 4:**
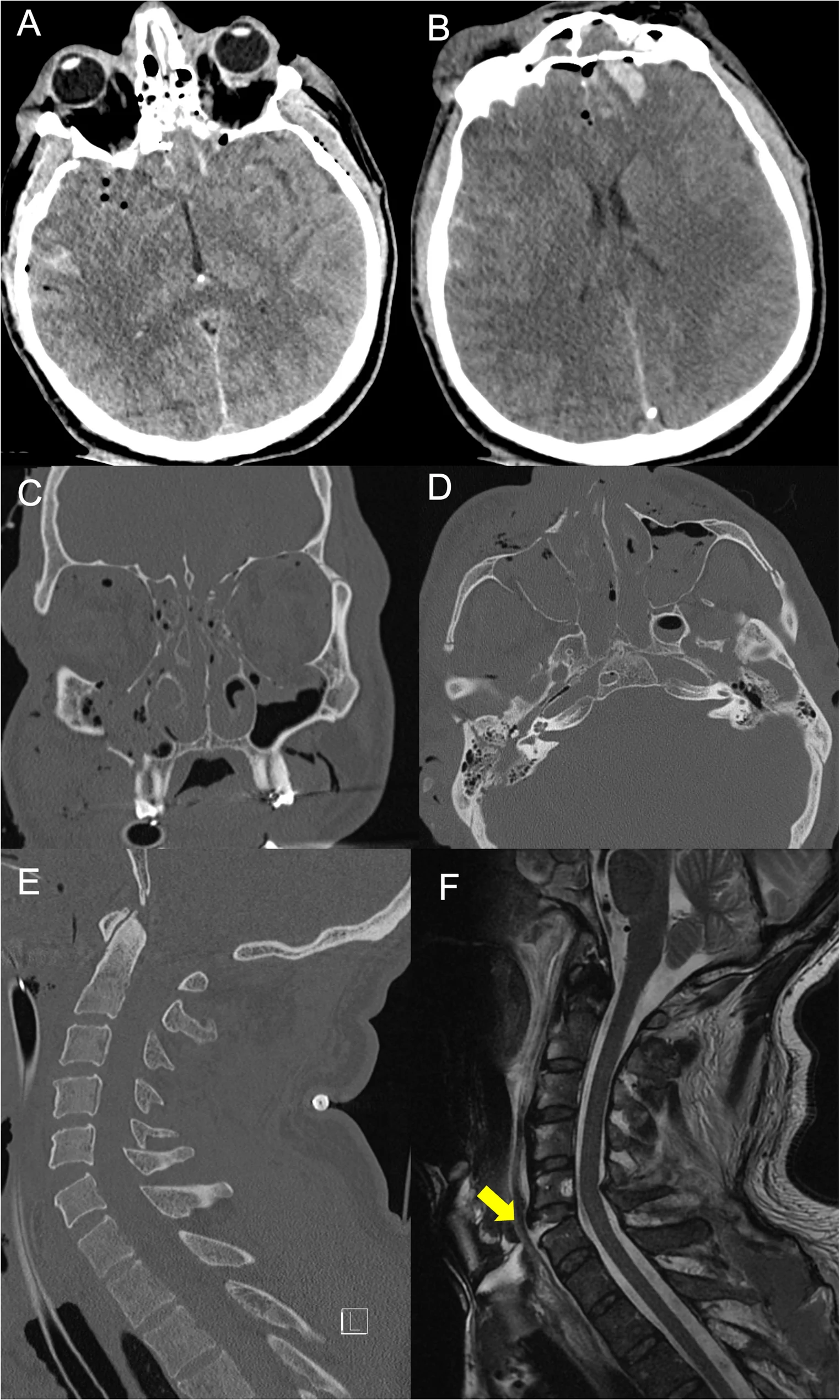
Imaging of a 54-year-old man following high-energy down-hill mountain biking trauma against trees. Head CT demonstrates bilateral frontal cerebral contusions with associated traumatic subarachnoid hemorrhage (**A**, **B**). Bone-window maxillofacial CT shows complex comminuted facial fractures (**C**, **D**). Cervical spine CT and MRI reveal a hyperextension mechanism injury (**E**, **F**), including MRI findings suggestive of early C5–C6 disco-ligamentous injury/distraction (yellow arrow, **F**)

From a radiological perspective, conventional radiographs are appropriate as the initial examination for suspected isolated fractures, whereas CT is essential when injury to the spine, pelvis, or thorax is suspected [14, 22]. Particular attention should be paid to fractures of the clavicle, radius, femur, and ribs, as well as spinal compression fractures, craniofacial trauma, and intracranial injury [4, 12–14] (Figs. 2, 3 and 4).

## Rock climbing: falls from height and axial loading

In contrast to DH-MTB, climbing trauma is primarily defined by vertical height, axial loading, secondary impact against rock, and occasional falling-object injury [15, 16]. Falls commonly generate axial loading and shear forces, with injury transmitted through the joints and skeleton [15–17]. Minor injuries include abrasions, bruises, and finger sprains, whereas moderate trauma includes fractures of the fingers, wrist, clavicle, ankle, or shoulder girdle [15, 16]. Severe events may result in cranial trauma, vertebral fracture, neurologic deficit, or internal organ injury [15, 16].

**Fig. 5:**
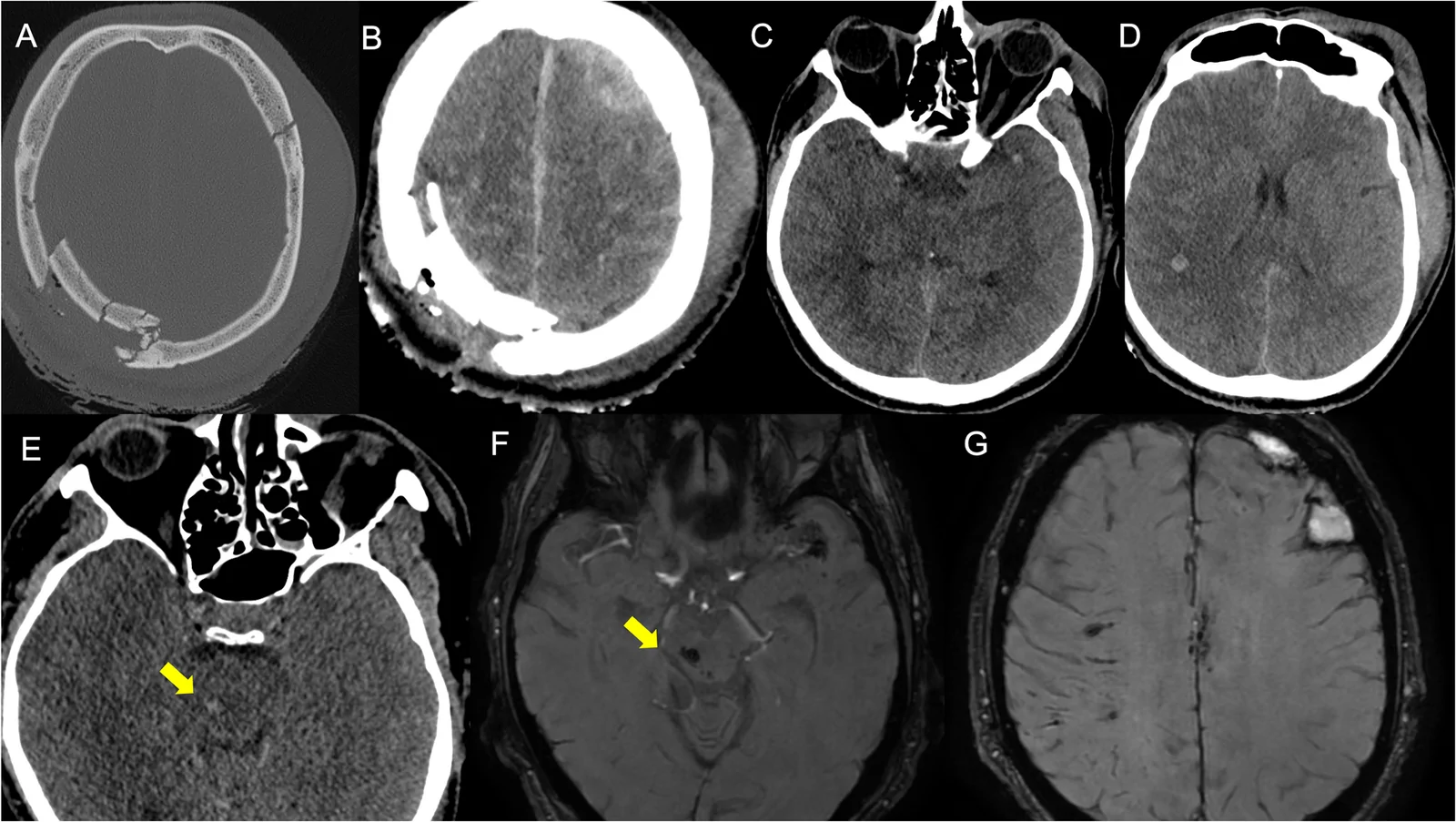
Imaging findings in a 55-year-old man who sustained severe cranial trauma from falling rocks while climbing. Bone-window CT demonstrates a comminuted skull fracture at the vertex, depressed in the right parietal region and extending toward the left frontal bone with involvement of the orbital roof (**A**). Axial CT images show extra-axial hematomas in the left frontal and right parietal regions, associated with traumatic subarachnoid hemorrhage (**B**), along with multiple traumatic intra-axial petechial hemorrhages in the left temporal subcortical region and bilateral frontal lobes (**B**, **C**, **D**, **G**), and a small hemorrhagic focus in the right mesencephalic region (yellow arrow, **E**). Susceptibility-weighted MRI confirms hemorrhagic traumatic axonal injury, with lesions involving the brainstem (yellow arrow, **F**) and lobar white matter (**G**)

**Fig. 6:**
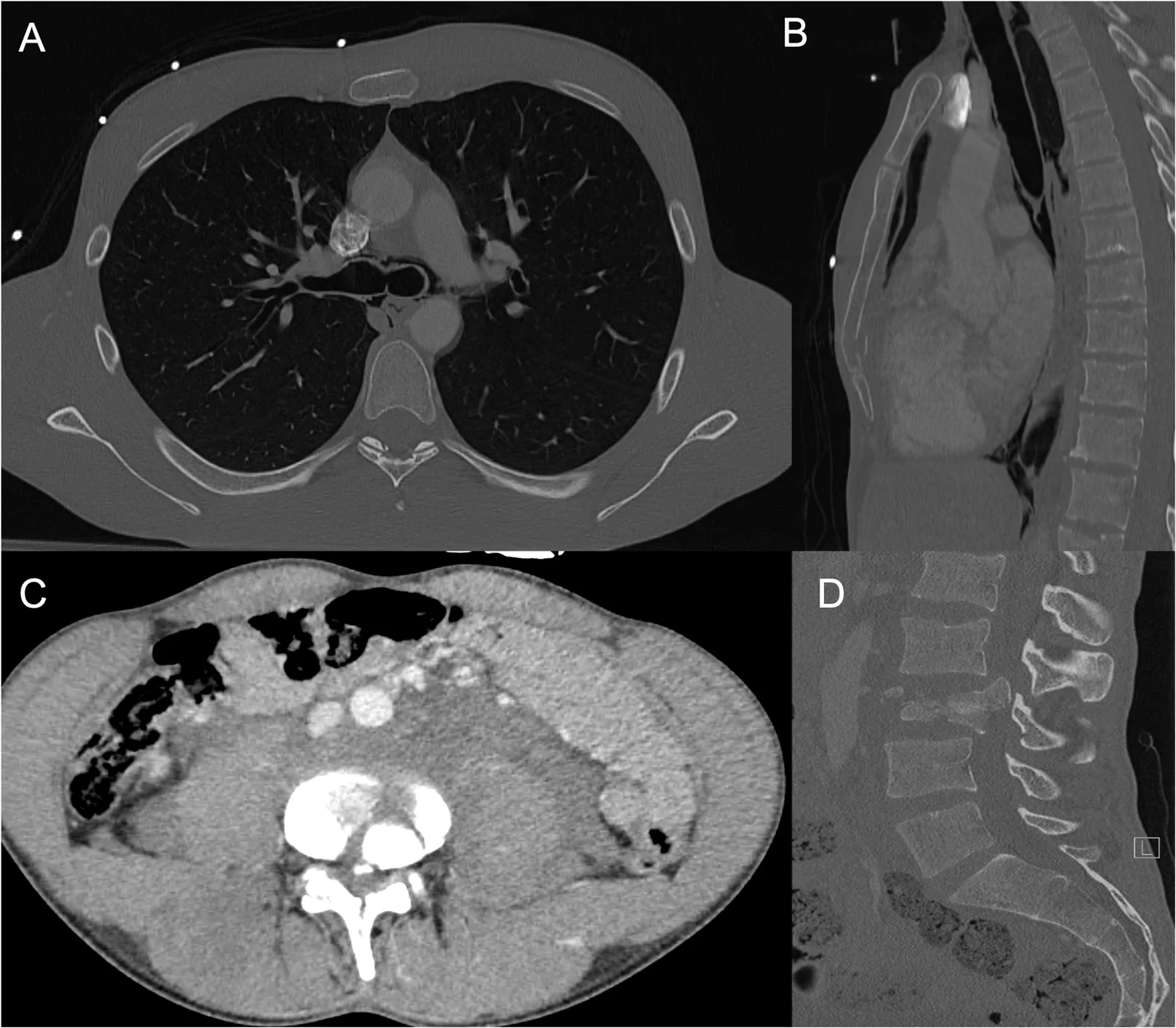
Contrast-enhanced CT findings in a 33-year-old man after a fall while climbing. Axial and sagittal chest CT images show traumatic pneumomediastinum (**A**, **B**) and sternal fracture (**B**). Abdominal CT demonstrates retroperitoneal hematoma (**C**), while sagittal lumbar spine CT shows an L3 burst fracture (**D**)

**Fig. 7:**
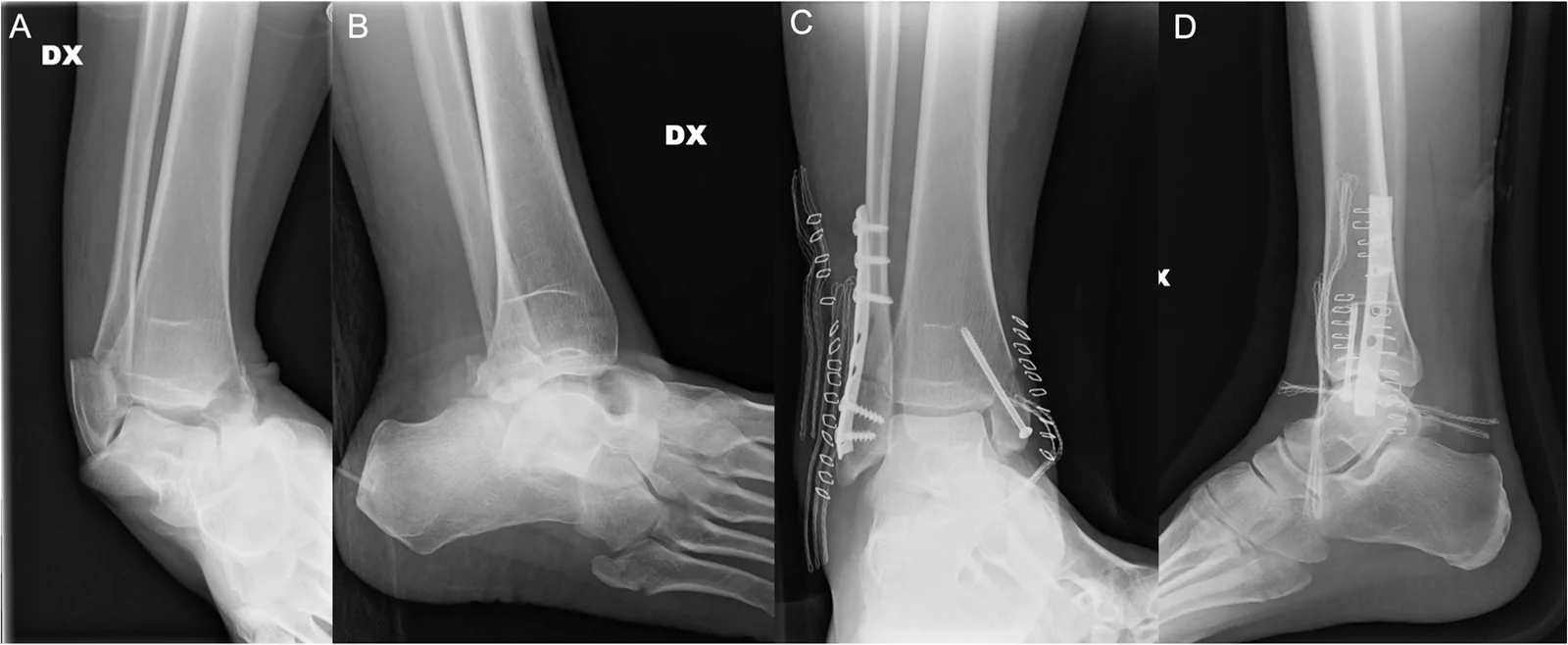
Plain radiographs of a 44-year-old woman who sustained trauma after a fall during rock climbing, with direct impact of the right foot against a rock spur. Initial anteroposterior and lateral radiographs demonstrate a talar fracture-dislocation associated with a displaced bimalleolar ankle fracture and a nondisplaced fracture of the base of the fifth metatarsal (**A**, **B**). Postoperative radiographs show restoration of articular alignment after urgent surgical reduction and internal fixation of the displaced fractures, performed to reduce the risk of talar osteonecrosis (**C**, **D**)

The upper limbs, especially the shoulder, clavicle, fingers, and forearm, are frequently involved, followed by the lower limbs and axial skeleton [15, 16]. Radiographs are useful for initial assessment of hands, wrists, ankles, and other appendicular fractures, whereas CT and MRI provide detailed evaluation of spinal fractures, especially at the thoracolumbar junction, pelvic injuries, and associated soft-tissue or neurologic damage [16, 22]. After axial loading, radiologists should actively search for coexisting calcaneal fractures, tibial plafond fractures, pelvic fractures, and vertebral compression or burst fractures, which may be missed if attention is directed only to the most obvious extremity injury [16, 17] (Figs. 5, 6 and 7).

## Paragliding: hard-landing and axial loading

Paragliding is an aerial sport in which the pilot flies a lightweight, foot-launched glider and controls direction and speed through brake lines [18, 19]. It shares the axial-loading pattern of falls from height, but the seated or semi-seated landing posture shifts the radiologist’s attention toward the thoracolumbar junction, pelvis, sacrum, and lower limbs. In alpine environments, hard landings and sudden wind shifts may generate substantial trauma, particularly to the spine, pelvis, and lower extremities [18, 19].

The main mechanisms of injury include controlled or uncontrolled hard landings, which transmit axial forces through the feet, ankles, knees, and pelvis. Twisting or torsional forces during loss of control may result in sprains, fractures, or ligamentous injuries [18, 19].

Injury severity is often moderate, with limb and rib fractures being common, but more severe trauma may include spinal injuries with or without neurologic deficit, thoracic or abdominal organ damage, and head trauma [18, 19]. The spine, particularly L1-L2 and the thoracolumbar junction because of the seated position, is among the most frequently affected sites, followed by the thorax, lower extremities, upper extremities, sacrum and pelvis, and the head and face, where intracranial hemorrhage, concussion, or skull fractures may occur [19].

**Fig. 8:**
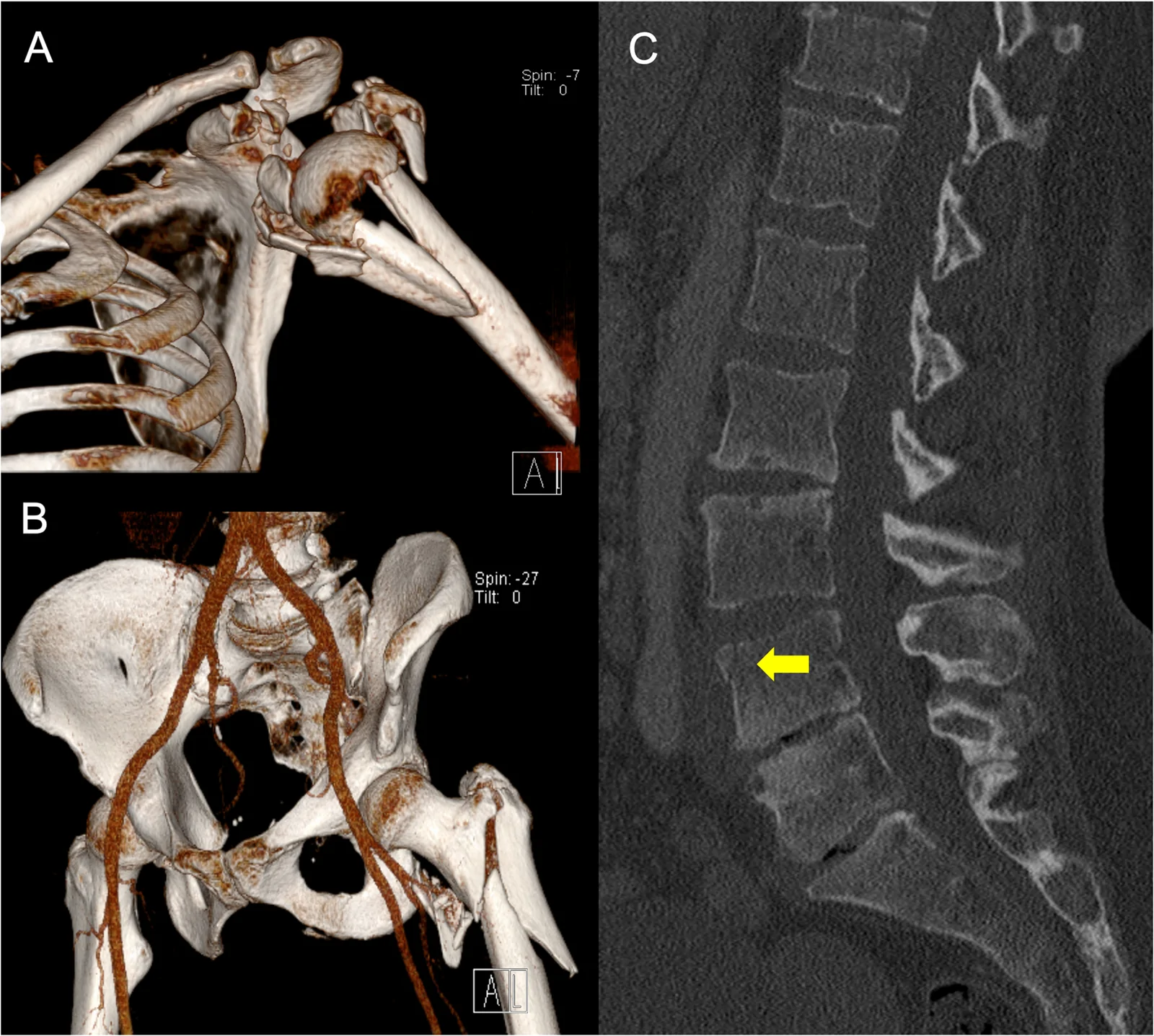
CT findings in a 54-year-old woman after a 10-meter fall during a paragliding accident. Three-dimensional CT reconstructions demonstrate a comminuted displaced fracture of the humeral head (**A**), a displaced pertrochanteric fracture of the femur, and a fracture of the left ischiopubic ramus (**B**). Sagittal lumbar spine CT shows a thin, nondisplaced fracture line at the anterosuperior corner of the L4 vertebral body (yellow arrow, **C**)

**Fig. 9:**
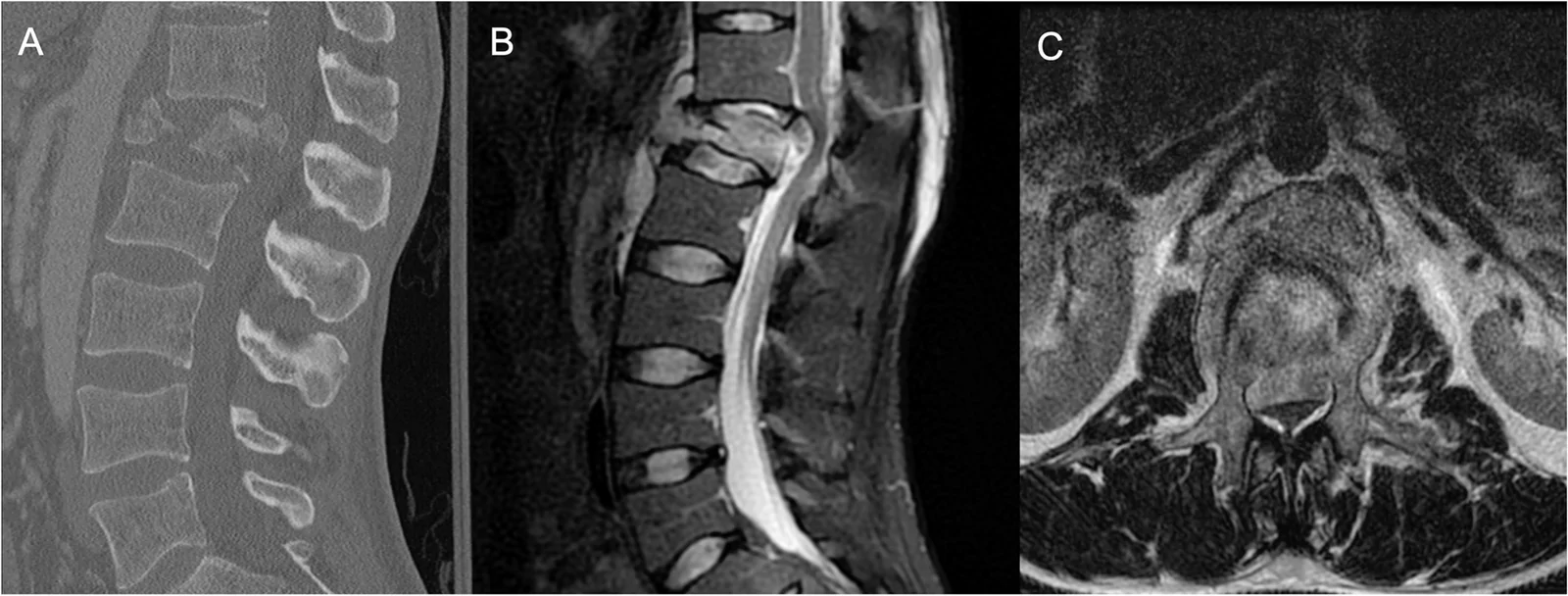
CT and MRI findings in a 34-year-old man with spinal trauma after a high-energy paragliding landing accident, with a fall from approximately 7 m. Sagittal CT and MRI images demonstrate an L1 burst fracture with retropulsion of a bony fragment into the spinal canal (**A**, **B**). Axial MRI confirms canal compromise with compression of the cauda equina nerve roots (**C**)

**Fig. 10:**
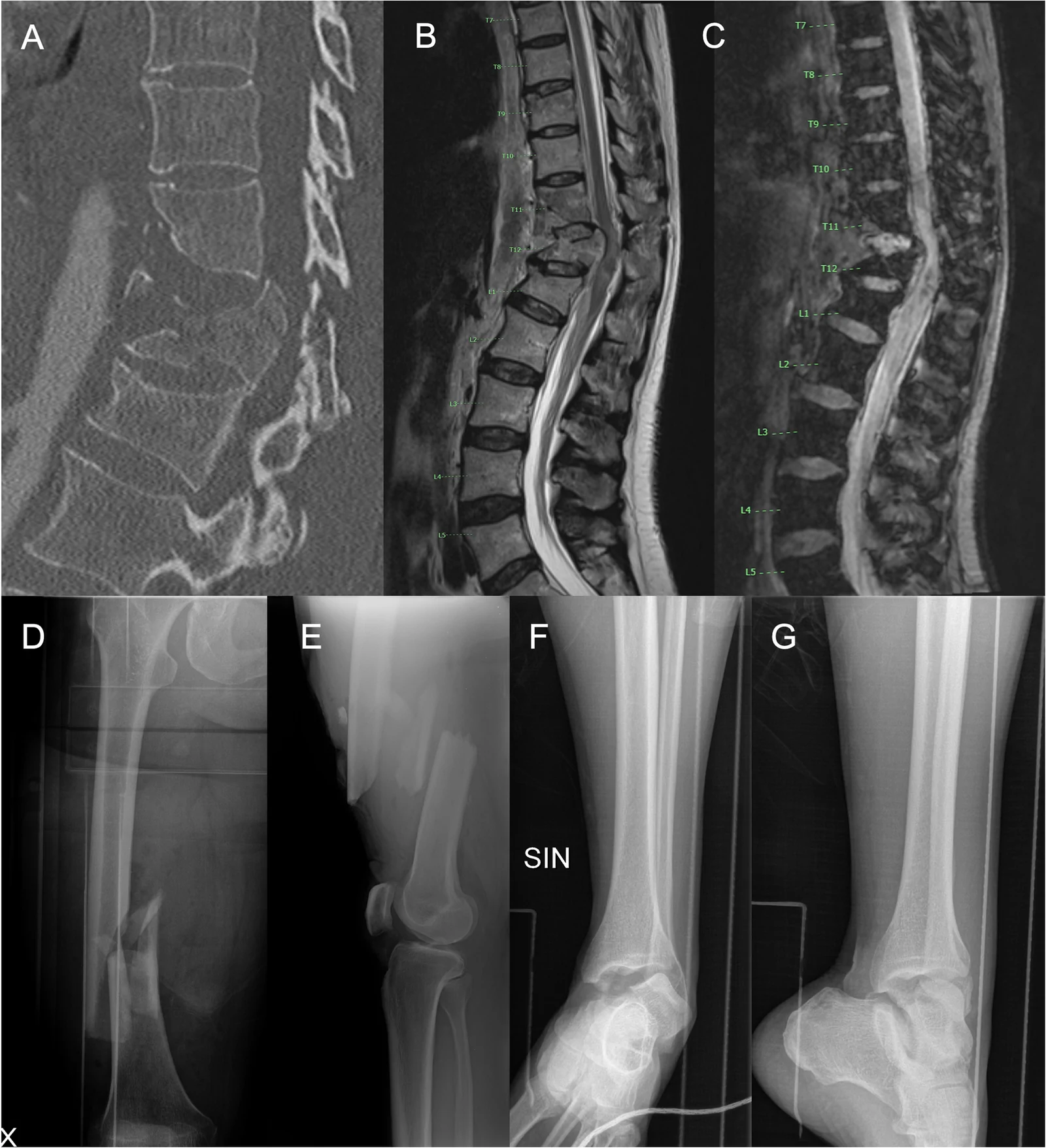
Multimodality imaging of a 55-year-old woman who sustained high-energy trauma during paragliding after loss of wing control, twisting of the canopy, and subsequent impact against avalanche barriers. CT and MRI demonstrate a T12 burst fracture with spinal canal compromise, spinal cord compression, and associated hematomyelia (**A**, **B**, **C**). Radiographs show an open, displaced, comminuted fracture of the right femoral diaphysis (**D**, **E**) and a left tibiotalar dislocation (**F**, **G**)

From an imaging standpoint, radiographs are mainly useful for the initial assessment of extremity trauma, particularly fractures of the ankles, feet, and lower legs, whereas CT and MRI are essential for evaluating spinal and pelvic injuries when axial loading is suspected [19, 22]. Particular attention should be paid to vertebral fractures, sacral fractures, and pelvic injuries, including acetabular fractures [18, 19] (Figs. 8, 9 and 10).

## BASE Jumping: high-velocity impacts and free-fall injuries

BASE jumping represents the highest-energy end of the aerial sports discussed in this pictorial essay. In alpine environments the activity most often involves free fall from cliffs or other fixed natural structures [20]. Injury severity is largely determined by exit height, free-fall velocity, parachute deployment, canopy control, landing posture, impact surface, and the presence of secondary impact against rock, trees, or the ground [4, 20].

The principal mechanisms of injury are failed or delayed parachute deployment, uncontrolled canopy flight, high-velocity landing, and impact against vertical or irregular surfaces [20]. These mechanisms may result in catastrophic trauma or death, and surviving patients should be considered at high risk for multiregional injury even when clinical findings initially appear localized [4, 20].

Landing feet-first is the posture most frequently associated with survivable falls from height, but it concentrates axial load through the lower limbs, pelvis, sacrum, and thoracolumbar spine [20]. As a result, radiologists should actively search for associated calcaneal, talar, tibial plafond, femoral, acetabular, pelvic ring, sacral, and thoracolumbar injuries, particularly burst fractures or fracture-dislocations with spinal canal compromise [11, 20, 22, 26].

More severe BASE-jumping trauma may include lumbosacral dissociation, spinopelvic dissociation, unstable pelvic ring disruption, spinal cord or cauda equina compression, thoracic injury, abdominal solid-organ injury, and major hemorrhage [4, 20, 22, 26]. Although these catastrophic patterns are less frequently encountered in routine emergency imaging because many such accidents are fatal at the scene, their recognition is essential in survivors because they may require urgent neurosurgical, orthopedic, interventional radiology, or trauma-team management [4, 20, 22, 26].

**Fig. 11:**
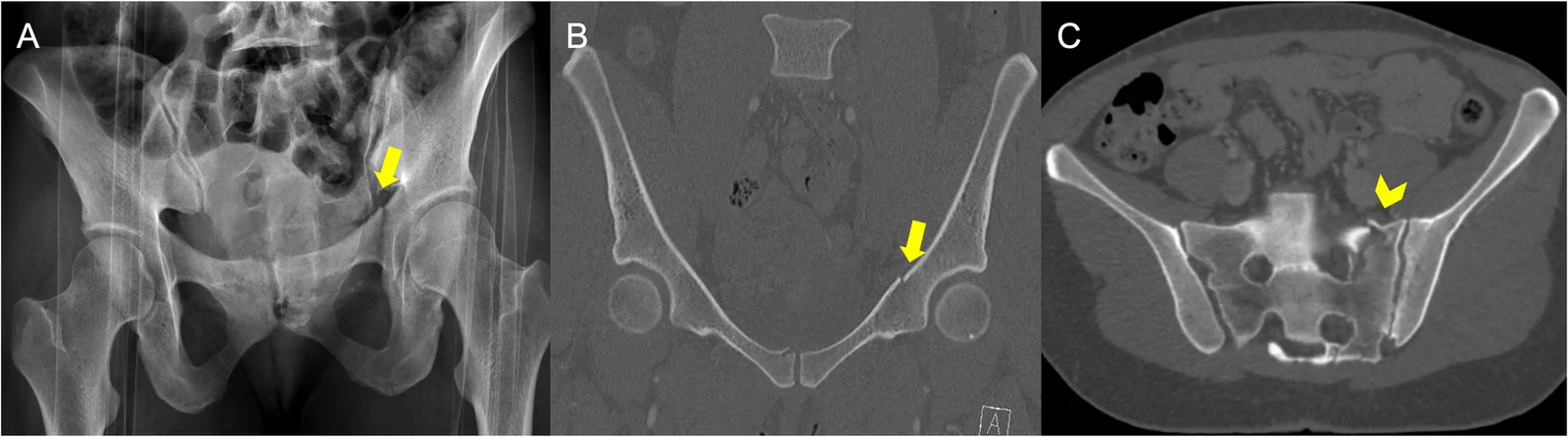
Imaging findings in a 36-year-old woman after a BASE-jumping accident, following parachute landing from approximately 10 m, initially on her feet and then in a seated position. Pelvic radiograph shows a fracture of the left iliopubic ramus (yellow arrow, **A**), confirmed on coronal CT reconstruction (yellow arrow, **B**). Axial CT demonstrates an associated fracture of the left sacral ala (yellow arrowhead, **C**)

**Fig. 12:**
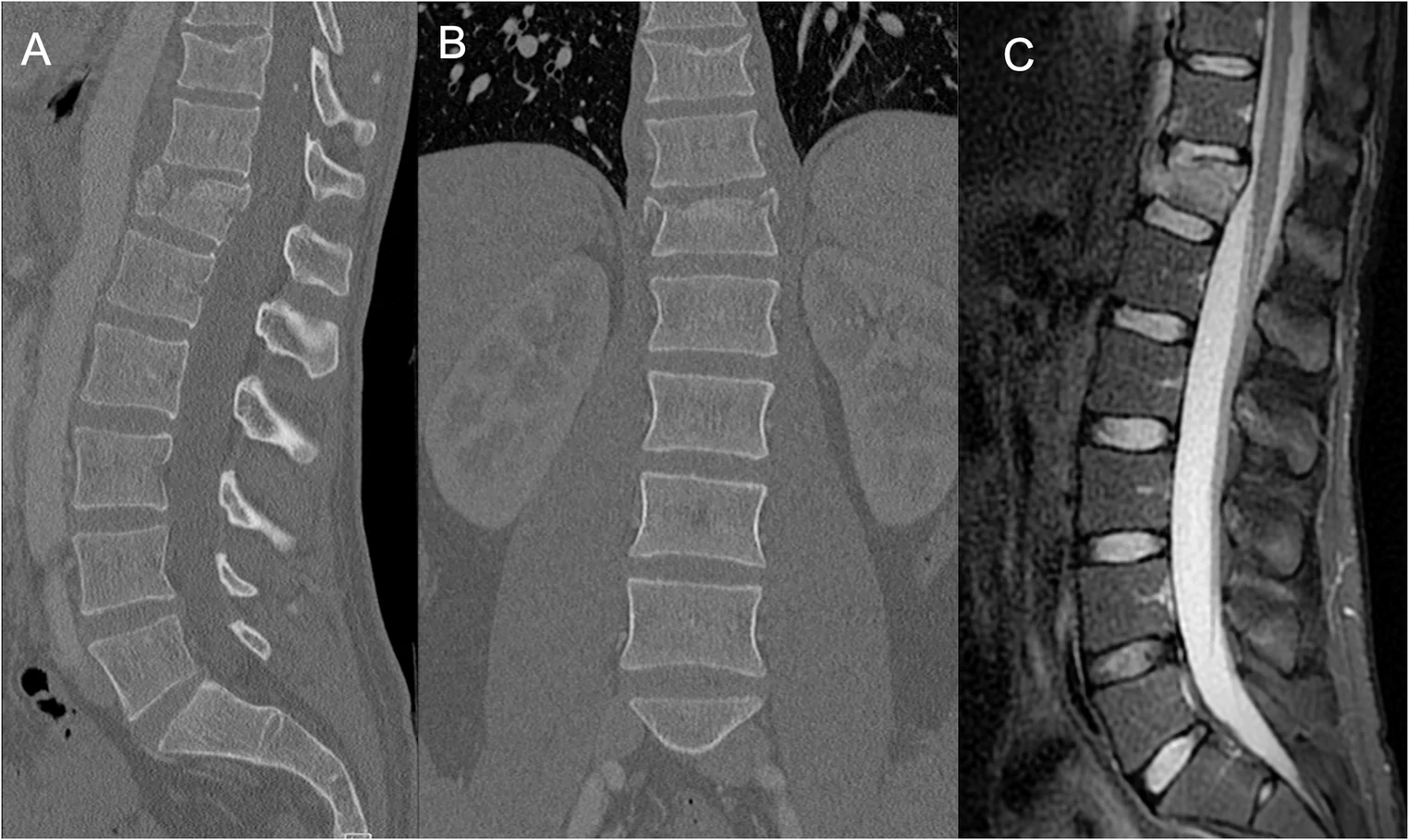
Imaging findings in a 29-year-old man who sustained trauma after a fall from approximately 5 m during the landing phase of a BASE jump. Sagittal and coronal CT reconstructions demonstrate a T12 burst fracture with vertebral body height loss and retropulsion of the posterior wall into the spinal canal (**A**, **B**). MRI further demonstrates the extent of the traumatic injury and spinal canal involvement (**C**)

Whole-body CT is therefore the preferred imaging modality in stable or stabilized patients after BASE-jumping trauma, particularly when the landing mechanism is high energy, the clinical examination is unreliable, or pain involves more than one body region [22–25]. Multiplanar spine and pelvic reconstructions should be systematically reviewed, and MRI should be performed after stabilization when neurologic symptoms, spinal canal compromise, ligamentous injury, epidural hematoma, or neural compression are suspected [22, 24, 26] (Figs. 11 and 12).

## Canyoning: water and rock impact trauma

Canyoning differs from aerial and cycling trauma because blunt impact is combined with water-related risks, including submersion, aspiration, hypothermia, and entrapment. Canyoning involves progression through rocky riverbeds, waterfalls, and narrow passages, exposing participants to a combination of blunt trauma from water impact, contact with sharp rock surfaces, and falls from height [5, 6]. 

The main mechanisms of injury include falls into water or onto wet rock, which may result in blunt trauma, fractures, or spinal injuries, as well as submersion and entrapment in confined spaces [5, 6]. These events can cause trauma related to water pressure or constriction and may be complicated by hypothermia or drowning [5, 6]. Lower-extremity trauma is the most common pattern of injury, followed by polytrauma, upper-extremity injuries, accidental hypothermia, spinal trauma, and drowning-related events [5, 6]. 

**Fig. 13:**
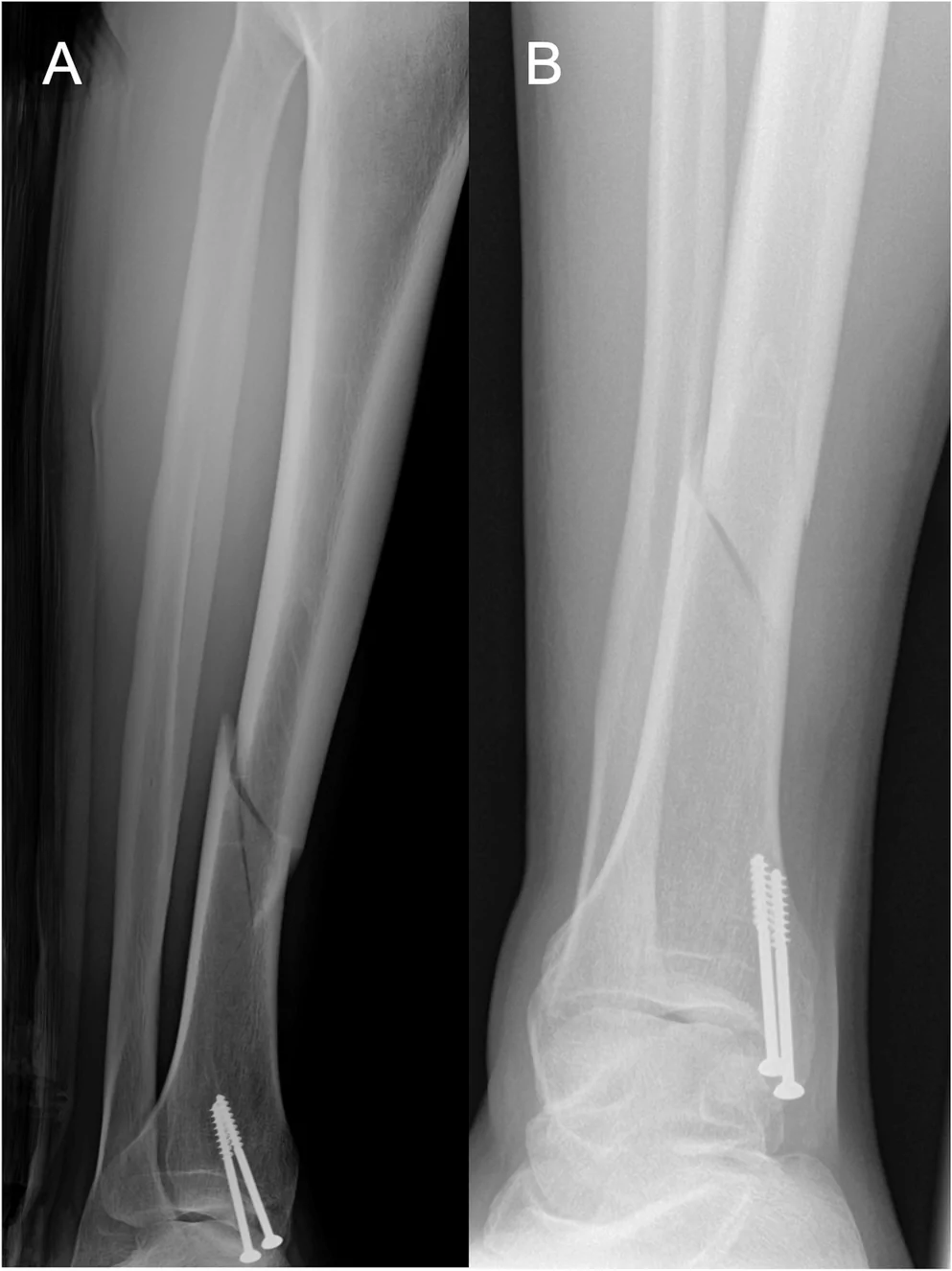
Plain radiographs of a 41-year-old man with trauma after slipping during canyoning. Anteroposterior and lateral views demonstrate a minimally displaced spiral fracture of the distal third of the tibial shaft (**A**, **B**). Note the sequelae of previous osteosynthesis of a tibial malleolar fracture, with placement of two metallic screws

**Fig. 14:**
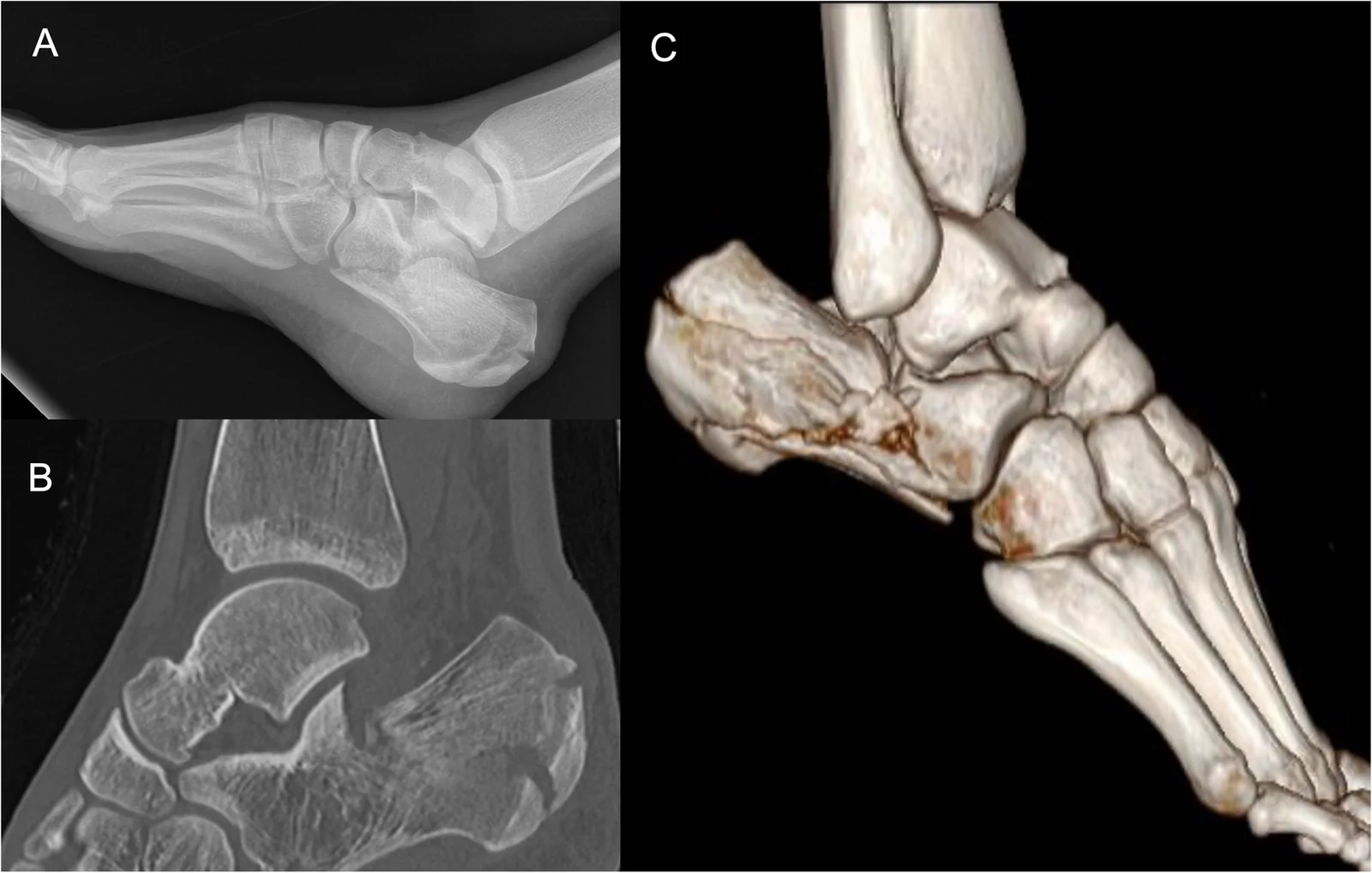
Imaging findings in a 21-year-old woman patient who sustained trauma during canyoning. Initial lateral radiograph shows a calcaneal fracture (**A**). Sagittal CT and three-dimensional CT reconstruction better demonstrate a displaced comminuted fracture of the calcaneus with extension into the subtalar articular surface (**B**, **C**)

From an imaging perspective, radiographs are useful for initial assessment of extremity fractures after falling onto rocks or into shallow water, whereas CT and MRI are essential for spine, chest, and abdominal injuries, especially after high-energy or underwater trauma [5, 6, 22]. Attention should be paid to rib fractures, pulmonary contusions, cervical and lumbar spine fractures, and fractures of the extremities [5, 6] (Figs. 13 and 14).

### Rafting: blunt trauma and torsional forces

Rafting involves navigating turbulent, fast-flowing water. Similarly to canyoning, it combines blunt trauma and water exposure, but injuries more often result from ejection, collision with rocks, capsizing, and near-drowning events [7, 8].

The main mechanisms of injury include blunt trauma from water impact, which may result in concussion, rib fractures, or extremity injuries. Direct collision with rocks or ejection from the raft can produce both torsional and blunt-force trauma, and drowning is an additional major risk in this setting [7, 8].

**Fig. 15:**
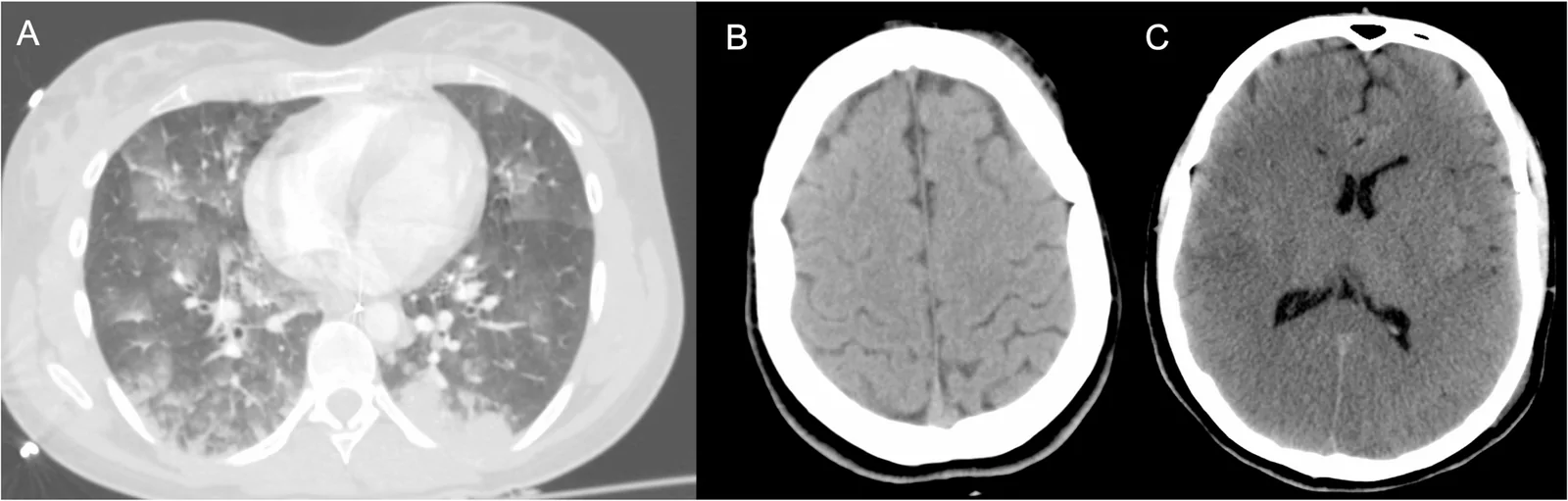
CT findings in a 23-year-old woman following a rafting accident with raft capsizing, head trauma, and near-drowning. Chest CT shows bilateral multifocal consolidative opacities, consistent with alveolar involvement related to drowning/aspiration injury (**A**). Head CT demonstrates a left frontal extracranial hematoma (**B**). The 24-hour follow-up head CT demonstrates diffuse cerebral edema without intracranial hemorrhage (**C**)

From a radiological perspective, conventional radiographs are appropriate for the assessment of suspected fractures involving the ribs or extremities, whereas CT and MRI are useful for evaluating head trauma, particularly after concussion, as well as possible spinal injury or pulmonary complications [8, 22]. Particular attention should be directed to rib fractures, extremity fractures, concussion-related intracranial findings when present, and cervical spine injuries [7, 8] (Fig. 15).

Table 2 summarizes the predominant injury patterns and imaging priorities in alpine summer ESs.

**Table 2:** Mechanism-based summary of the predominant acute injury patterns and imaging priorities in extreme alpine summer sports. CT, computed tomography; MRI, magnetic resonance imaging

Sport	Mechanism of injury	Predominant injury pattern	Do not miss
Down-hill mountain biking	Lateral falls, over-the-handlebar crashes, abrupt deceleration, impact against obstacles	Upper-extremity fractures are most common, with possible rib, facial, spinal, and pelvic injuries in high-energy trauma	Clavicular, radial, femoral, and rib fractures, spinal compression fractures, craniofacial trauma, and intracranial injury.
Rock climbing	Falls from height, axial loading, impact against rock or ground, swinging injuries, rockfall	Upper-limb trauma is frequent, with possible calcaneal, ankle, pelvic, thoracolumbar, and head injuries after high-energy falls	Calcaneal, tibial plafond, pelvic, and vertebral compression fractures.
Paragliding	Hard landing, axial loading through the lower limbs, twisting forces during loss of control	Thoracolumbar and pelvic trauma predominate, often associated with lower-extremity, thoracic, and head injuries	Vertebral, sacral, and acetabular fractures, rib fractures, and intracranial injury.
BASE jumping	High-velocity impact after free fall, failed or inadequate parachute deployment	Severe trauma involving the lower limbs, trunk, and spine, often with multiregional injury	Lower-extremity fractures, lumbosacral and pelvic injury, spinopelvic dissociation, and thoracoabdominal organ damage.
Canyoning	Falls, impact against rock, forceful water impact, submersion, entrapment	Lower-extremity injuries are most common, but spine, thoracic, and multiregional trauma may occur	Rib fractures, pulmonary contusions, cervical and lumbar spine fractures, and extremity fractures.
Rafting	Water impact, collision with rocks, ejection from the raft, torsional trauma, drowning	Rib, extremity, cervical spine, and head injuries are the main traumatic patterns	Rib fractures, extremity fractures, cervical spine injury, concussion-related intracranial findings, and pulmonary complications.

## Conclusion

In alpine summer ESs, the variety of injury mechanisms requires a tailored emergency radiology approach. Prompt and accurate diagnosis with radiography, CT, and MRI is essential. Understanding typical injury patterns guides care, supports timely intervention, and may reduce long-term complications.

## Data Availability

Data sharing is not applicable to this article as no new data were created or analyzed in this study.
